# Doing Systematic Literature Reviews With Artificial Intelligence Tools: What, Why, and How

**DOI:** 10.1093/geroni/igaf122.1472

**Published:** 2025-12-31

**Authors:** Bo Xie, Kimia Tuz Zaman, Wordh Ul Hasan, Juan Li

**Affiliations:** The University of Texas at Austin, Austin, Texas, United States; North Dakota State University, Fargo, North Dakota, United States; North Dakota State University, Fargo, North Dakota, United States; North Dakota State University, Fargo, North Dakota, United States

## Abstract

Systematic reviews are indispensable to evidence-based practice but require considerable time and effort, particularly in screening large numbers of studies. Recent advances in large language models (LLMs) offer a promising avenue to reduce this burden through partial automation of the process. We present a two-stage multi-agent framework that leverages LLMs—to supplement human expertise—in conducting systematic literature reviews of AI interventions for older adults. In Stage 1, specialized LLM agents independently assess titles and abstracts against seven predefined criteria—including population age (≥65 years), AI focus, care setting, study design, outcome measures, older adult participation, and empirical evidence. An aggregator LLM agent then synthesizes these evaluations into a composite inclusion score. In Stage 2, a set of specialized LLM agents extracts structured data from full-text articles based on targeted research questions addressing technology types, outcome effectiveness, user acceptance, and ethical considerations. The extracted data are then merged into a comprehensive dataset that supports both qualitative and quantitative synthesis. Although our multi-agent system significantly reduces the time and labor traditionally required for manual screening and data extraction—while enhancing consistency through task specialization—human oversight remains essential for resolving ambiguous cases and making necessary adjustments. While the complete results of applying this framework will be reported elsewhere, this paper focuses on the method, its rationale, and its potential to accelerate evidence synthesis in the face of an ever-growing biomedical literature base. This innovative, scalable approach exemplifies how hybrid human–AI methods can advance the rigor and timeliness of systematic reviews.

